# Traumatic Radius Pseudoarthrosis in Neurofibromatosis Type 1: All Treatment Options in One Patient

**DOI:** 10.7759/cureus.34024

**Published:** 2023-01-20

**Authors:** Çağdaş Pamuk, Ülker Moralar

**Affiliations:** 1 Orthopaedics and Traumatology, Silivri Anadolu Special Hospital, Istanbul, TUR

**Keywords:** microsurgery, neurofibromatosis 1, bone transplantation, pseudarthrosis, radius fractures

## Abstract

Although congenital pseudoarthrosis of the radius is a rare case, it has been reported many times in the literature in the past and it has been shown that it can be associated with neurofibromatosis type 1. However, traumatic radius pseudoarthrosis has never been reported before. In this case report, all treatment options were applied to the same patient over a four-year period and the results were reported. A two-year-old boy had a left radius diaphysis fracture after a simple fall, and bone union could not be achieved despite the application of cast immobilization, internal fixation grafting, and electrical stimulation in an external center. He was admitted to our hospital when he was six years old. A plain X-ray image and computed tomography scan showed that he had radius pseudoarthrosis and also he was diagnosed with neurofibromatosis type 1 on genetic analysis. The patient underwent wide resection of the segment with pseudoarthrosis and free vascularized fibula grafting. Bone union was achieved in the third postoperative month.

## Introduction

Neurofibromatosis type 1 (NF-1) is a hereditary disease with 1:3000 incidences worldwide. Although common clinical findings are neurofibromas and cafe au lait spots, sphenoid dysplasia, anterolateral tibial bowing or tibial pseudoarthrosis, and kyphoscoliosis can be seen in association with NF-1. Less common orthopedic findings are congenital forearm pseudoarthrosis and more often congenital radius pseudoarthrosis [1].

Various treatment modalities for congenital radius pseudoarthrosis associated with NF-1 have been reported in the previous literature [2-5]. Although there are cast immobilization, autologous grafting with internal or external fixation, and electrical stimulation among the treatment options, acceptable bone union rates were found to be low in cases with NF-1 gene defect [6]. Therefore, in recent years, free vascularized fibula graft (FVFG) application has become widespread in this group of patients, allowing wide segment resection and good results with proper union [6-8].

In this case, we reported the long-term results of the treatment of severe deformity and pseudoarthrosis, which developed as a result of the failure of all conservative and surgical treatment options, in a pediatric patient with NF-1 who had traumatic, unlike congenital radius pseudoarthrosis, with wide resection and FVFG application.

## Case presentation

**Figure 1:**
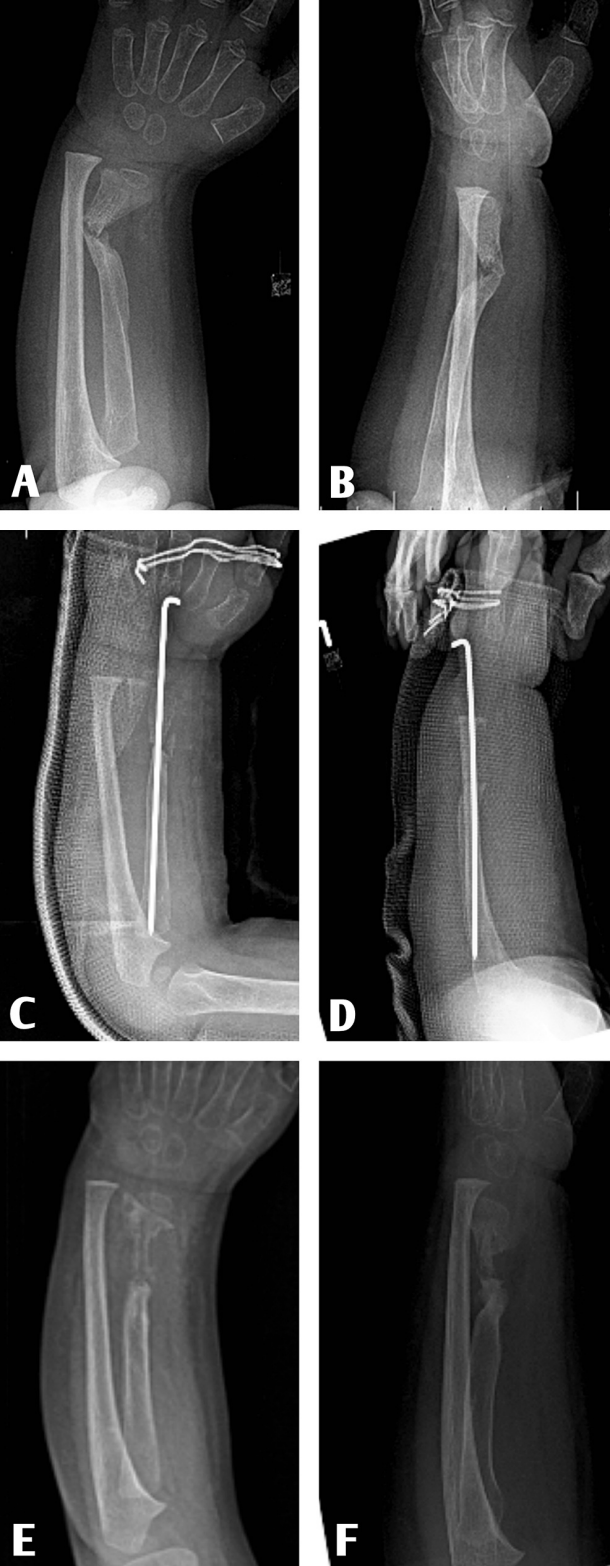
Interventions performed on the patient at two years of age. A, B. X-ray after cast immobilization. C, D. X-ray after internal fixation + grafting. E, F. X-ray after electrical stimulation for one month.

A two-year-old male patient was treated in an external center with plaster immobilization for two months after a simple fall, and as a result of the development of radius pseudoarthrosis, cortico-cancellous grafting from iliac wing and internal fixation were applied in another center. The patient was followed up for two more months and there was no sign of union, and the rod was removed and electrical stimulation treatment was applied (Figure 1).

**Figure 2:**
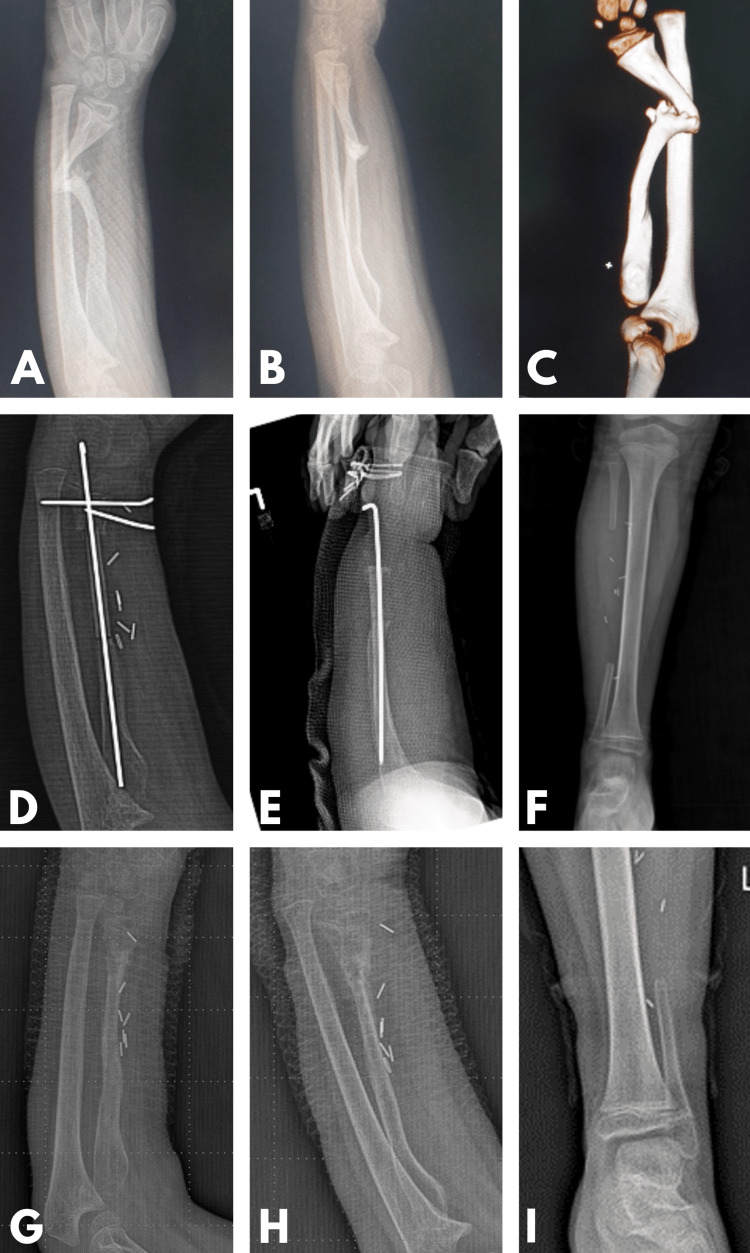
Evaluations and interventions when the patient reached six years of age A, B, C. Initial evaluation X-ray and three-dimensional (3D) computed tomography images. D, E, F. X-ray images of forearm and cruris after vascularized fibula operation. G, H, I. X-ray images of forearm and cruris six months after vascularized fibula operation.

Despite this, the patient, whose union could not be obtained, was followed up for four years without any additional intervention and was admitted to our clinic at the age of six with severe deformity of the radius, pseudoarthrosis, and shortness (Figure 2).

No deformity was observed in the ipsilateral ulna, but it was observed that there was a 1 cm shortening when compared to the contralateral ulna. In the general systemic examination of the patient, multiple cafe au lait spots were observed and the diagnosis of NF-1 was made as a result of the genetic analysis. Wrist extension was 20 degrees, flexion 40 degrees, forearm pronation was full, and supination was 10 degrees on admission. Radial and ulnar artery patency was found to be normal in the Doppler ultrasound.

**Figure 3:**
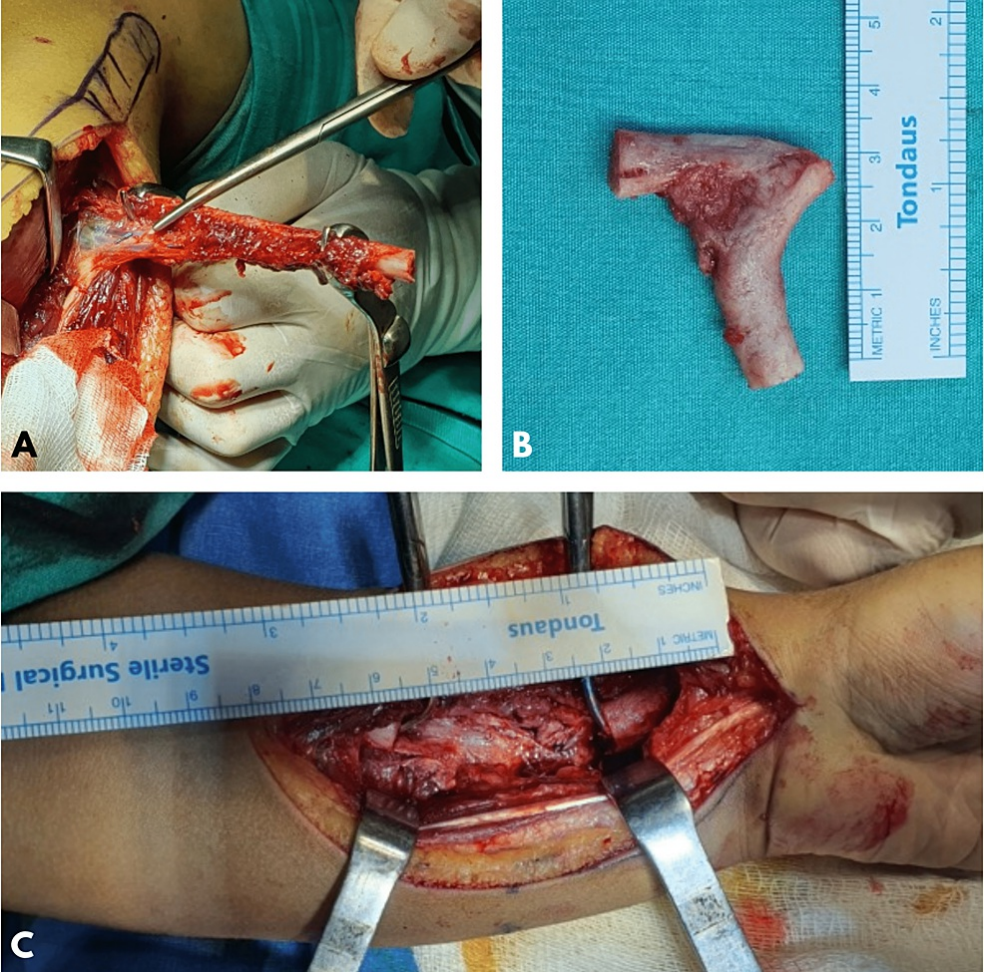
Intraoperative images of the patient. A. Controls of vascularized fibula graft before proximal pedicle separation. B. The resected bone segment with pseudoarthrosis. C. The appearance of the bone defect in the forearm after resection.

Complete resection of the deformed pseudoarthrosis segment and FVFG application were planned for this patient. The deformed segment was completely removed using the volar Henry approach (Figure 3). It was removed to the limit of viable bone. Viability in the bone was determined by noticing the bleeding.

A 10 cm segment of the ipsilateral fibula was taken as free vascularized with the peroneal artery and vena comitantes. Since a segment longer than 10 cm was left distal to the fibula, no additional fixation was performed on the ankle. A 6 cm FVFG segment was intramedullary fixed with a k-wire from the distal radius segment with appropriate tension. To prevent rotation of the distal segment, two transverse k-wires were placed just proximal to the epiphysis (Figure 2).

End-to-end anastomoses of fibula peroneal artery with recipient site radial artery and vena comitantes with vena cephalica were performed. The patency of the anastomoses was tested and confirmed. The authors, who are specialists in orthopedics and traumatology, performed all surgical procedures in the order mentioned.

Postoperatively, 100 mg acetazolamide was given per-orally once a day for 14 days. During hospitalization, Dextran-40 was administered intravenously at 0.4 ml/kg/h for three days. The patient was recommended to use 500 mg of first-generation cephalosporin (cefalexin) twice a day for one week.

**Figure 4:**
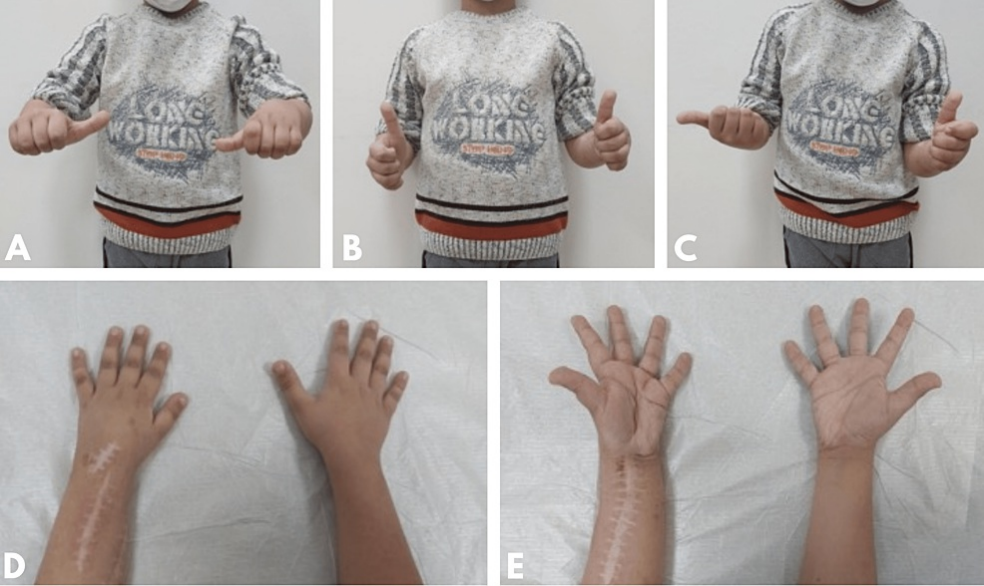
Postoperative follow-up photographs of the patient. A. Pronation, B. neutral, C. supination, D. comparative forearm dorsal view, E. comparative forearm volar view.

Radiographic union was achieved in the proximal part at week 4 and in the distal part at week 7 (Figure 2). After an 18-month follow-up, there was 1 cm of radial shortening and radial translation of the wrist, and the patient's wrist flexion-extension and pronation movements were limited to full supination at 10 degrees (Figure 4). No problems were observed in the leg and gait from which FVFG was harvested.

## Discussion

Although 67 cases with congenital radius pseudoarthrosis with NF-1 were reported in the previous literature, according to the very best of our knowledge, this case is the first case with traumatic radius pseudoarthrosis [6]. At the same time, it is a special case that all conservative and surgical options are applied in the same patient with radius pseudoarthrosis and successful results were achieved only with FVFG application. Among the treatment options for radius pseudoarthrosis, cast immobilization, grafting with internal fixation, and electrical stimulation applications have been defined in previous studies and there is no consensus on their results yet [9]. In our case, all of these applications were applied and a bony union could not be achieved. In terms of cast immobilization, it was reported in the past literature that union could be achieved in only one case, while unsuccessful results were reported in eight patients [6,10]. In the application of internal or external fixation and grafting, the union was reported in 58.8% of the cases, and unsuccessful results were reported in the other half. Similar results have been reported in electrical stimulation applications [4-6].

With the widespread use of microsurgery, FVFG, which allows wide-segment resection, has now become applicable in many centers. Successful results have been reported in many cases requiring extensive resection, with or without concomitant syndromic conditions [11-14]. However, no case of nonunion developing as a result of FVFG application to congenital radius pseudoarthrosis with NF-1 patients has been reported before. A successful union was also achieved in our case. FVFG has been shown to be the most valuable treatment modality for pseudoarthrosis, an NF1 disease.

Another issue in the FVFG application is the fixation technique. External fixation, intramedullary rod, and plate-screw fixations are mostly applied osteosynthesis techniques. Since the risk of deformity development was found to be high in pseudoarthroses close to the distal segment before, plate and screw applications were recommended [7]. In our case, transverse K wires were used with intramedullary rod application for control of the distal segment, and no complications developed. Beris et al. reported that they applied FVFG in the second session after reaching the appropriate length in order to prevent the development of radius shortness by applying distraction with an external fixator in the first stage [6]. However, since this application was not suitable for the deformity in our case, we preferred the single-stage FVFG application. It was planned to follow up the x-ray images of the patient regularly every six months and check whether a "radial club hand"-like deformity has developed. If no additional deformity develops, the ulnar shortening procedure can be performed when he reaches adulthood.

## Conclusions

Radius pseudoarthrosis with NF-1 is a difficult clinical condition to treat and there is a high possibility that complications may develop in the follow-up. Unsuccessful results with cast immobilization, internal or external fixation, and grafting in congenital or traumatic pseudoarthrosis with NF-1 have been observed in our case and in previous reports. We think that wide segment resection and FVFG application can be applied primarily in this group of patients.
